# Prehabilitation in Major Surgery: An Evaluation of Cost Savings in a Tertiary Hospital

**DOI:** 10.3390/jcm14072460

**Published:** 2025-04-03

**Authors:** Natalia Mudarra-García, Fernando Roque-Rojas, Visitación Izquierdo-Izquierdo, Francisco Javier García-Sánchez

**Affiliations:** 1 Research Nursing Area, Hospital Universitario Ramón y Cajal, Instituto Ramón y Cajal de Investigación Sanitaria (IRYCIS), 28040 Madrid, Spain; nmudarra@enf.ucm.es; 2 Nursing Department, Faculty of Nurse, Phisiotherapie and Podology, University Complutense of Madrid, 28040 Madrid, Spain; 3 Surgical Prehabilitation Unit, Hospital Universitario Infanta Cristina, Instituto de Investigación Sanitaria Hospital Puerta de Hierro Segovia Arana (IDIPHISA), 28040 Madrid, Spain; fernando.roque@salud.madrid.org (F.R.-R.); visitacion.izquierdo@salud.madrid.org (V.I.-I.); 4 Medical Department, Faculty of Medicine, University Complutense of Madrid, 28040 Madrid, Spain

**Keywords:** prehabilitation, advanced practice nurse, surgery, postoperative complications, perioperative care

## Abstract

(1) **Background**: Prehabilitation programs improve patients’ functional capacity before surgery by enhancing physical activity, nutrition, and psychological well-being, thereby reducing postoperative complications, hospital stays, and readmissions. We propose a centralized model led by an advanced practice nurse and internist to minimize consultations and reduce costs. (2) **Methods**: We studied 211 patients in a tertiary hospital in Madrid, with 135 enrolled in the centralized prehabilitation program and 76 in standard care (control). We compared complications, hospital stays, blood transfusions, and consultations, estimating costs using public pricing from Madrid’s healthcare authorities. (3) **Results**: The centralized model significantly reduced blood transfusions (*p* = 0.014), postoperative complications (*p* < 0.001), and hospital stays (*p* = 0.004), leading to annual savings of EUR 593,453.00. (4) **Conclusions**: A centralized surgical prehabilitation model decreases complications, hospital stays, readmissions, and consultations compared to standard care, significantly reducing healthcare costs.

## 1. Introduction

Since the Danish surgeon Henrik Kehlet introduced the fast-track concept in the 1990s, it has been incorporated with increasing acceptance by all healthcare professionals involved directly or indirectly in the surgical process. The perioperative changes introduced by this concept have allowed for better postoperative outcomes, which ultimately translate to quicker recovery to the patient’s basal state [1]. This application of fast-tracking brings evident benefits, but, currently, the concept alone is insufficient due to the increased number of comorbidities, greater frailty, and increased life expectancy of the population. For these reasons, we need better preoperative optimization of patients’ basal state, beyond minimizing possible surgical complications. To achieve this, it is necessary to improve their functional state (physical, nutritional, and psychological), as this is one of the factors involved in poor postoperative evolutions and one on which we can act [2].

Factors such as malnutrition, frailty, sarcopenia, and anemia are particularly relevant in patients with head and neck cancer, pancreatic neoplasms, and other digestive tumors, significantly impacting surgical tolerance and prognosis. Severe malnutrition, common in these malignancies, increases the risk of infections, delays wound healing, and results in poorer response to adjuvant therapy. Sarcopenia and frailty impair the response to surgical stress, contributing to the loss of functional independence and prolonged hospital stays. Preoperative assessments, such as muscle mass measurement and physical performance tests, can help identify high-risk patients. Preoperative anemia, frequently observed in oncologic patients, is associated with an increased need for transfusions, perioperative fatigue, and worse postoperative outcomes. Strategies such as iron supplementation or erythropoietin administration can be key in its management.

Based on these factors, prehabilitation is a program designed to improve the patient’s functional capacity before surgery by addressing three aspects: physical activity, proper nutrition, and reduction in anxiety and frustration. Several published studies support the improvement in presurgical functional condition with prehabilitation programs, which may include specific interventions such as exercise, education, smoking cessation, nutritional assessment and education, psychological support, as well as optimization of comorbidities and their treatments [3,4,5,6,7,8].

This preparation requires active participation from the patient, and, for this, it is necessary for them to understand the significance of their effort [9,10].

There are many models of how to carry out surgical prehabilitation. In some hospitals, such as the Clinic of Barcelona, this program is led by anesthesiologists and includes nursing staff from anesthesia, physiotherapists, nutritionists, and psychologists. Additionally, it is supported by other specialists like surgeons, pulmonologists, and cardiologists, who are involved throughout the surgical process. In these programs, the initial assessment is performed by the anesthesiologist, and the patient is referred to different professionals as needed [11]. Other models are managed by the surgery department and refer the patient to various specialists according to their needs [12]. With these models and others, the patient’s condition optimized, but this requires multiple consultations.

The results of prehabilitation in abdominal surgery are also diverse. Authors like Boden I. et al. [13] and Martin D. et al. [7,14] have asserted that these programs reduce postoperative complications and hospital stays, in addition to being reliable and safe, significantly reducing annual hospital costs [7,13,14,15]. Another study conducted at the Infanta Cristina University Hospital, where prehabilitation consultation was led by nurses and internists, evidenced that, with this model, the quality of life and functionality of patients were improved, and postoperative complications, readmissions, hospital stays, and healthcare costs were reduced [16].

Within this framework, the main goal of the present study was to evaluate cost savings at a tertiary hospital following the presented prehabilitation model. As secondary objectives, postoperative complications, length of hospital stay after the surgical intervention, and blood requirements used in the immediate and distant postoperative periods were analyzed.

## 2. Materials and Methods

### 2.1. Study Design

An observational, descriptive, comparative pre–post study was conducted at the University Hospital Infanta Cristina in Parla (Community of Madrid, Spain) on patients over 18 years old who underwent scheduled major surgery. The surgical prehabilitation program, led by an advanced practice nurse and supported by an internist, was applied to the intervention group (which received functional, clinical, and psychological interventions) and compared with the group receiving standard preoperative care.

### 2.2. Inclusion—Exclusion Criteria

The surgical prehabilitation program began in early 2020 for all patients on the surgical waiting list scheduled for major surgery, except those undergoing emergency surgery, hospitalized patients, or patients of the Department of Otolaryngology.

This retrospective review of patient records covered surgeries performed between 1 January 2019, and 31 December 2023. All participants had previously been included on the surgical waiting list for major surgery, including oncological procedures (digestive, urological, and gynecological surgeries) and non-oncological procedures such as orthopedic surgeries (primary hip and knee arthroplasty) and gynecological surgeries (hysterectomies). Patients who were transferred to other hospitals or canceled their surgery were excluded from this study.

### 2.3. Sample Size

Regarding patient recruitment, the control group included patients who underwent surgery in 2019, while the intervention group included patients who participated in the prehabilitation program and underwent surgery between 2020 (with an interruption due to COVID-19), 2021, 2022, and 2023.

### 2.4. Studied Variables

The following clinical and demographic variables were collected: sex, age, and oncological disease (yes or no).

In addition, descriptive variables were recorded before surgery, such as the number of consultations performed to optimize the patient. Postoperative variables related to clinical outcomes, including complications, the number of red blood cell units required, and the average hospital stay duration until discharge, were also analyzed.

Another key aspect of data collection was the cost savings generated following the implementation of the surgical prehabilitation program.

### 2.5. Intervention

The intervention group followed the preoperative intervention program for between 15 and 30 days before the procedure.

The process began at the moment the responsible surgeon placed the patient on the surgical waiting list, generating a referral to the Surgical Prehabilitation Unit, along with laboratory tests associated with the program to detect deficiencies, anemia, and other conditions such as diabetes and dyslipidemia, which may affect the perioperative and late postoperative periods.

**Figure 1:**
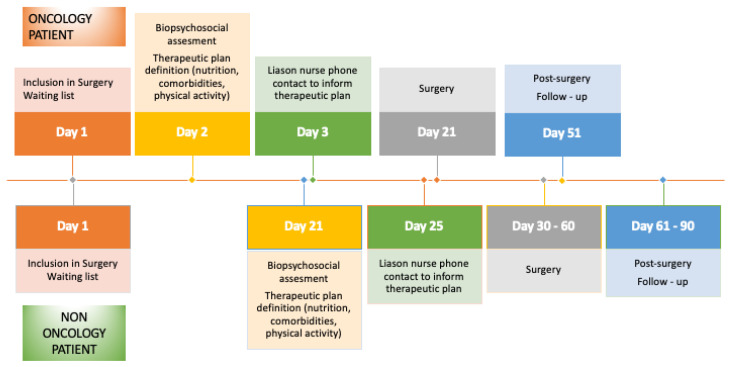
Prehabilitation process.

If the patient had an oncological condition, they were evaluated by an advanced practice nurse (APN) within 48 h of inclusion, prior to the anesthesia assessment, to allow for maximum optimization time, given that surgery occurred within 30 days. If the patient was scheduled to receive neoadjuvant therapy before surgery, the evaluation took place at the beginning of the treatment to prevent the deterioration caused by oncological therapy before surgery. This was due to the limited time frame available before the surgical intervention. For non-oncological surgeries, the timeline was more flexible, and evaluations were conducted 21 to 30 days before surgery (Figure 1).

**Figure 2:**
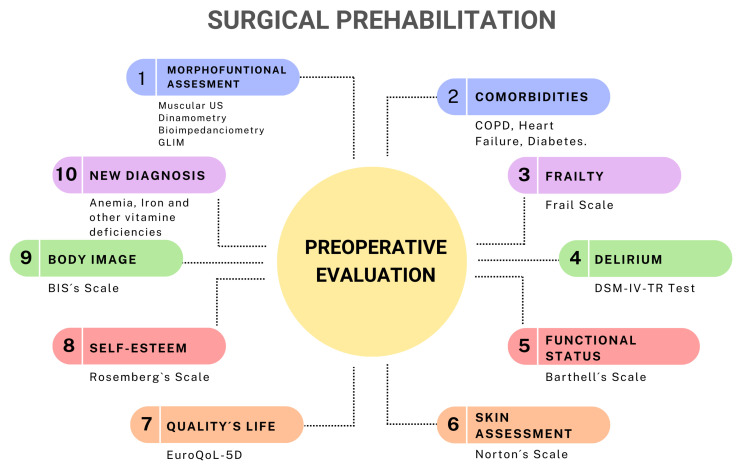
Ten phases of prehabilitation program.

Once the patient was assessed in the prehabilitation program, they underwent comprehensive screening, including a bio-psychosocial assessment (Figure 2), which consisted of

- Nutritional assessment using GLIM criteria.
- Laboratory tests including C-reactive protein (CRP), albumin, prealbumin, lymphocytes, and cholesterol.
- Multifunctional assessment, including BMI, and rectus femoris muscle measurements via ultrasound, along with complete bioelectrical impedance analysis (BIA) including fat mass, muscle mass, and total body water using phase angle (PA) estimation.

Additional laboratory tests assessed hemoglobin levels, iron profile, vitamin B12, and folic acid to identify anemia (preoperative hemoglobin < 13 g/dL) and vitamin D levels, which correlate with muscle contraction.

The functional assessment included the following:

- Six-meter walk test and handgrip dynamometry to evaluate muscle contraction;
- Degree of dependence and frailty assessed using the Barthel index and, later, the FRAIL scale;
- Pulmonary capacity assessment and optimization using a respiratory incentive device, which was provided directly during consultation to increase pulmonary capacity.

Additionally, psychological evaluation was performed by

- Measuring self-esteem using the Rosenberg Self-Esteem Scale;
- Assessing body image perception using the Body Image Scale (BIS) questionnaire;
- Determining quality of life with the EuroQoL 5D test.

If the patient presented with low self-esteem (Rosenberg < 25 points) and/or poor quality of life (EuroQoL 5D > 5 points), they were referred to a psycho-oncologist for further assessment and intervention.

### 2.6. Cost-Saving Estimation

To estimate the cost savings in euros for the hospital resulting from the implementation of the program between 1 January 2020, and 30 December 2022, the inclusion visit, the visit the day before surgery, and the visit one month after surgery were considered. For the effectiveness/efficiency analysis in this study, the following cost-saving indicators were selected: hospital stay savings, the number of consultations saved (due to avoided referrals to other specialists), the number of complications prevented (including re-operation), and, finally, the number of red blood cell concentrate units saved. This allowed us to calculate the corresponding economic savings.

Complications often result in additional surgical intervention or pharmacological treatment. Taking this into consideration, the estimated cost per complication was based on DRGs 252.2 (abdominal/gastrointestinal procedure complications).

These unit prices in euros are based on Order 1975/2023 of 29 December from the Madrid Health Department, which establishes the public prices for healthcare services and activities in the Community of Madrid [17].

### 2.7. Statistical Analysis

A descriptive analysis was performed for all study variables to examine their distribution. Categorical variables are described using the percentage associated with each possible response option, while quantitative variables are summarized using the mean, standard deviation, and range.

To assess the normality of the data, the Kolmogorov–Smirnov test for a single sample was applied to the quantitative variables. For comparisons between variables and hypothesis testing, the following statistical methods were used: the chi-square test for categorical variables; Student’s *t*-test or ANOVA for normally distributed quantitative variables; and the Mann–Whitney U test or Kruskal–Wallis test for quantitative variables that did not follow a normal distribution. Additionally, odds ratios (ORs) were calculated to estimate the relationships between binary dichotomous variables.

## 3. Results

**Figure 3:**
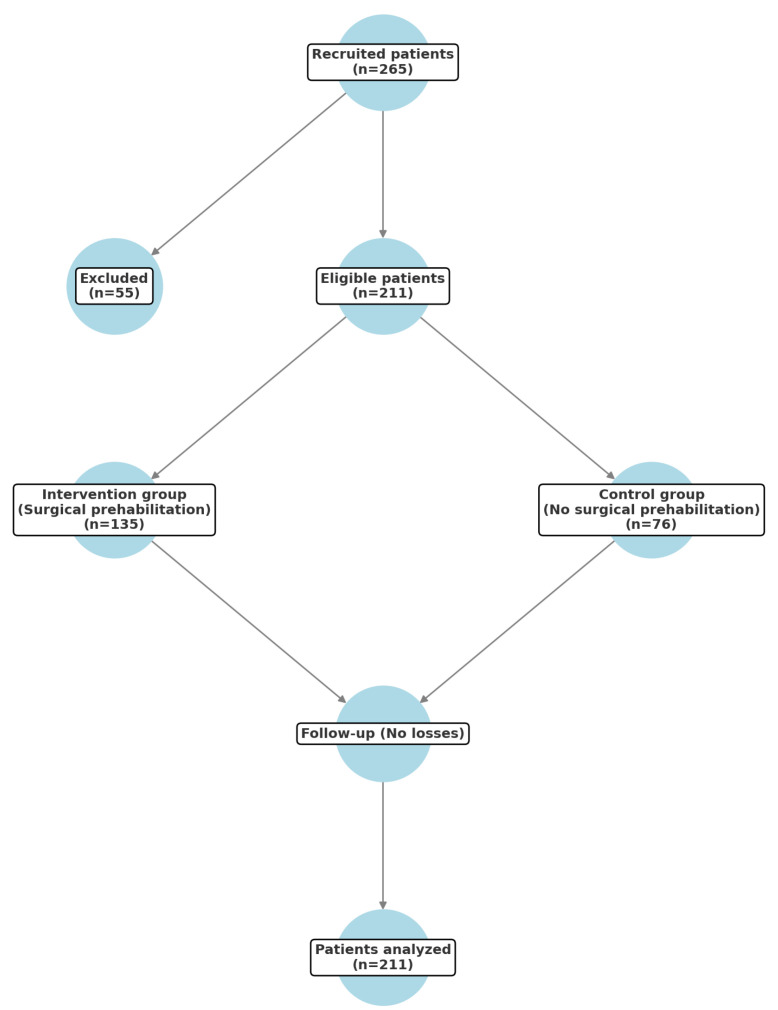
Recruitment and selection diagram.

A total of 265 patients were recruited for this study, of whom 55 were excluded because they were transferred to other hospitals to expedite their surgery due to administrative reasons. The remaining 211 patients were randomized: 135 to the group that received optimization before surgery (intervention group) and 76 to the group who did not receive it (control group) (Figure 3).

The sample consisted mostly of men (58.5%), with a mean age of 65.67 years (Table 1). A total of 88.6% were oncology patients, and only 14.4% had received neoadjuvant therapy. On the other hand, 11.4% were non-oncological patients.

Regarding complications, statistically significant results were obtained (*p* < 0.001), with the control group experiencing more complications after surgery (52.6% versus 25.0% in the intervention group). The OR for complications based on whether optimization was received before surgery was 3.434 (95% CI (1.890; 6.241).

The intervention group required fewer blood transfusions than the control group (90.1% versus 75%, *p* = 0.014) [OR = 3.128; 95% CI (1.445; 6.772)]. Similarly, the intervention group received fewer red blood cell concentrates than the control group (0.80 versus 2.24, *p* < 0.001). The length of hospital stay and readmissions were higher in the control group compared to the intervention group (*p* = 0.004, *p* = 0.014, respectively) (Table 2).

### 3.1. Costs

For this study, the calculation included the 135 patients in the intervention group and the 76 patients in the control group.

#### 3.1.1. Hospital Stay Savings

**Figure 4:**
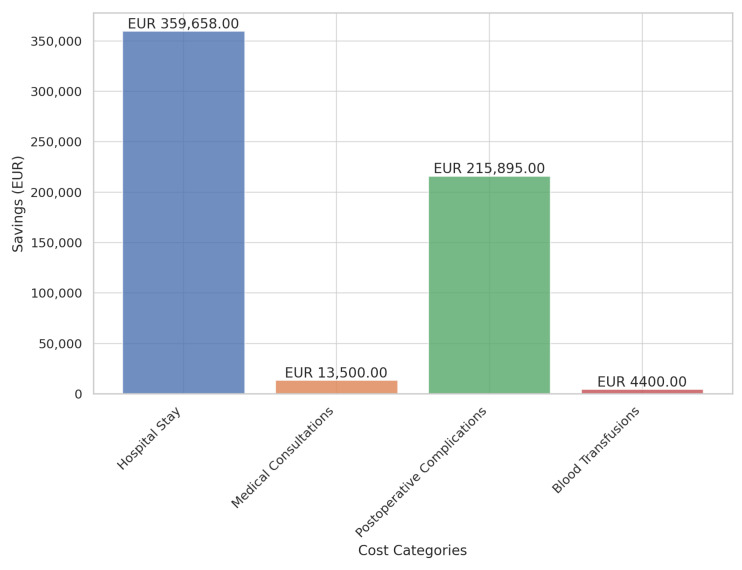
Total saving of the surgical prehabilitation program.

Based on the obtained data, the average hospital stay was 8.34 days for patients who participated in the prehabilitation program versus 11.63 days for those in the control. This results in a total savings of 444.15 hospital stay days, which was equivalent to 53 potential admissions for gastrointestinal, urological, gynecological, hepatobiliary, or pancreatic surgery, with an average cost per admission of EUR 6786.00. Therefore, the total savings amounted to 53 potential discharges/admissions × EUR 6786.00 per discharge, totaling EUR 359,658.00 (Figure 4).

#### 3.1.2. Reduction in Preoperative Consultations

The reduction in the number of consultations following the establishment of the surgical prehabilitation unit was estimated at 21.3%. This translated to a savings of 59 consultations for the 135 patients in the intervention group compared to the control group. Given that each consultation costed EUR 75.00, the total savings amounted to EUR 4425.00.

#### 3.1.3. Reduction in Postoperative Complications

In the control group, 52.6% of patients experienced postoperative complications compared to 25.0% in the intervention (prehabilitation) group. This corresponded to avoiding postoperative complications in 37 patients. The estimated cost per complication, based on DRG 252.2 (abdominal/gastrointestinal procedure complications), was EUR 5835.00. Calculated over 37 patients, this resulted in a total savings of EUR 215,895.00 (Figure 4).

#### 3.1.4. Savings on Blood Transfusion Requirements

Regarding the need for red blood cell transfusions, 25.0% of the control group required transfusions compared to 9.9% in the intervention (prehabilitation group). This meant that transfusions were avoided in 20 patients, with an average of two blood-derived units per patient (costing EUR 110.00 per unit), resulting in total savings of EUR 4400.00 (Figure 4).

#### 3.1.5. Total Program Savings

**Figure 5:**
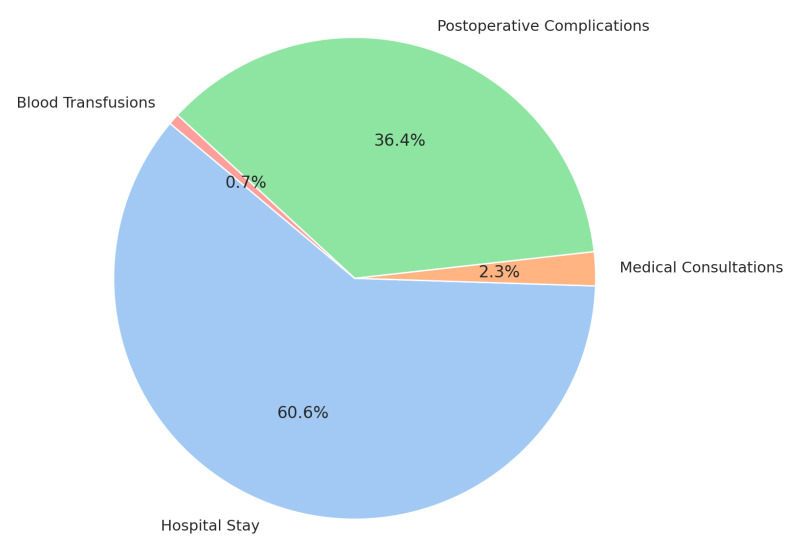
Distribution of total savings from a prehabilitation program.

The total savings from the program amounted to EUR 593,453.00 for the sample of 135 patients (Table 3). See percentage at Figure 5.

## 4. Discussion

Patient preparation before major surgery for people with cancer between 10 and 21 days prior to the operation and patients without cancer between 30 to 60 days prior (Figure 2) had many beneficial effects, including enhancing nutritional status with hyperproteical supplements and carbohydrate-rich shakes for those with malnutrition, increasing lung capacity using an incentive spirometer, a physical exercise program, and reducing anemia and comorbidities through medical treatment [18,19,20,21,22]. This study showed that adequate preoperative preparation significantly reduced the complication rate in oncological interventions (*p* < 0.001). Patients undergoing this regimen not only had shorter hospital stays (*p* = 0.004) but also fewer readmissions (*p* = 0.014) compared to the control group. Thanks to this system, a total of 53 potential admissions were avoided, resulting in savings of EUR 359,658.

A relevant aspect is the number of visits required before surgery. National prehabilitation models (GERM Group) require intervention from multiple specialists (hematologists, endocrinologists, cardiologists, pulmonologists, physiotherapists, etc.) and a higher number of consultations, increasing systemic costs and impacting patient quality of life due to time spent on multiple appointments, averaging five visits per patient and delaying prehabilitation start. At the Hospital Infanta Cristina of Parla, the model we implemented, following IMPRICA and belonging to the GERM group, centralizes the coordination of advanced practice nursing with an internist, allowing optimization to begin within 48 h from inclusion in a surgery waiting list, requiring only one patient visit, thus improving quality of life and reducing hospital costs. This represents a reduction of 59 consultations, translating into a benefit of EUR 4425.00.

According to evidence from Schack et al. and the Spanish multimodal rehabilitation group, the risks of mortality and complications are higher for patients with untreated anemia [23]. As suggested in the study, early optimization of patients’ hemoglobin levels reduced postoperative complications. Our study showed that those who did not undergo surgical prehabilitation had a 3.43 times lower risk of experiencing postoperative complications compared to those who did. This led to postoperative complications being avoided in 37 patients, resulting in savings of EUR 215,895.00.

**Figure 6:**
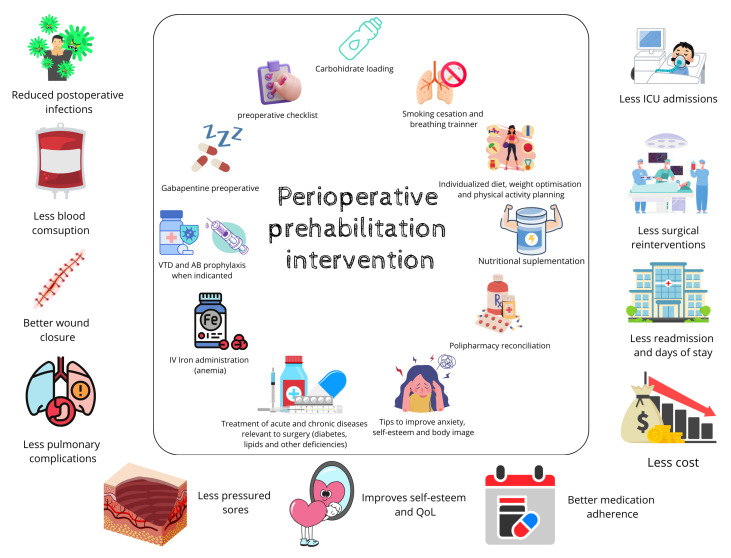
Perioperative intervention.

With optimal hemoglobin levels, although the patient could experience bleeding during surgery, postoperative hemoglobin levels were higher than if blood products were not administered when levels are below 13 g/dL. This helped prevent decompensation in patients with heart failure, exacerbation of chronic kidney disease, or delays in postoperative rehabilitation caused by acute anemic syndrome (Figure 6). Additionally, delayed hospital discharge could increase the risk of nosocomial infection. In summary, this translated into a reduced need for blood (25.0% in the control group versus 9.9% in the intervention group), shorter postoperative stays, fewer complications for the patient, and cost savings for the system. This resulted in savings of 40 units of blood products, generating a benefit of EUR 4400.00.

This was reflected in improved 30-day mortality rates, as described by Schack et al. in their series [23].

The aforementioned surgical prehabilitation program, in terms of cost-effectiveness, translated into savings of EUR 593,453.00 (savings of EUR 4395.95 per patient), as evidenced in this article. This study indicates the increase in savings and supports the findings of Rombey et al. [24]. Despite the positive results in the meta-analysis, our program at the Infanta Cristina University Hospital yielded even greater savings.

Advanced practice nurses in surgical prehabilitation clinics play a fundamental role in optimizing patients’ health status before surgery, ensuring individualized and evidence-based care. Their competencies include leadership in care planning, resource management, and coordination of the multidisciplinary team, ensuring continuity of care and patient safety, as described by Mudarra et al. [16] Through comprehensive assessment, they identify specific needs, optimize comorbidity management, and promote self-care to improve functionality and quality of life. Additionally, they provide personalized health education, monitor treatment adherence, and facilitate communication between different levels of care. Their work contributes to the sustainability of the healthcare system by reducing hospitalization time, postoperative complications, and readmissions, thereby optimizing resource utilization. Prehabilitation clinics enhance patient and caregiver satisfaction while strengthening professional training through continuous education and the generation of knowledge via research. Their positive impact on cost reductions, improved safety, and the efficiency of surgical care highlights the importance of their role within a multidisciplinary team. In this way, advanced practice nurses not only improve clinical and economic outcomes but also foster more effective, patient-centered, and humanized healthcare, in line with what was stated by Assolari et al. [25].

## 5. Study Limitations and Future Perspectives

The main limitations of our study are related to the external validity of the findings. First, the cost-savings analysis was conducted in a single hospital within the Spanish public healthcare network, which may limit the generalizability of the results to other centers with different organizational structures, care protocols, or funding sources.

Additionally, our study did not include certain surgical pathologies, such as head and neck neoplasms or cardiac surgery, among others. The exclusion of these groups prevented us from assessing the impact of surgical prehabilitation in people with different recovery profiles and preoperative needs, which may affect the applicability of our findings to a broader population.

Future studies should expand the analysis to multiple hospital centers not only within Spain but also in other countries with different healthcare financing models, including those with non-public or unsubsidized healthcare systems. This would allow for an evaluation of the economic and clinical feasibility of surgical prehabilitation in contexts with differences in funding structures and treatment accessibility, providing a more global perspective on its impact on healthcare system efficiency.

## 6. Conclusions

This study confirmed that implementing a surgical prehabilitation model led by an advanced practice nurse and an internist significantly reduced postoperative complications, decreased hospital length of stay, and optimized healthcare resource utilization in a tertiary hospital. Patients who participated in the prehabilitation program had a 3.43 times lower risk of postoperative complications, a reduced need for blood transfusions, and a significant decrease in hospital readmissions compared to those who did not undergo prehabilitation.

From an economic perspective, this model proved to be highly efficient by reducing unnecessary preoperative consultations and optimizing the care process, allowing for better coordination across different levels of care. Additionally, the improved preoperative preparation facilitated a faster return to patients’ baseline functionality, leading to more efficient postoperative recovery and reducing the overall impact of surgery on quality of life.

Moreover, this model reinforces the crucial role of advanced practice nursing in perioperative care management, highlighting its leadership in interdisciplinary coordination, patient education, and evidence-based decision making.

## Figures and Tables

**Table 1:** Sociodemographic and clinical variables.

		Control (n = 76)	Study (n = 135)	Total (n = 211)	*p*-Value
Age mean (SD)		67.88 (10.15)	64.43 (12.06)	65.67 (11.50)	0.370
Sex	Men n (%)	53 (69.7%)	73 (54.1%)	127 (58.6%)	0.030
	Women n (%)	23 (30.3%)	62 (45.9%)	84 (39.1%)	
Oncological patient	Yes n (%)	67 (88.2%)	119 (88.8%)	186 (88.6%)	0.527
	No	9 (11.8%)	16 (11.2%)	25 (11.4%)	
Neoadjuvant therapy	Yes	10 (13.23%)	20 (15.2%)	30 (14.4%)	0.694
	No	66 (86.8%)	115 (84.8%)	181 (85.6%)	

**Table 2:** Cost-saving table specified by units and concepts, as well as individualized costs with a prehabilitation program led by an advanced practice nurse.

	Units Earned	Cost per Unit (EUR)	Total Earned (EUR)
Hospital stay length	53 days	6786.00	359,658.00
Medical visits	180	75.00	13,500.00
Adverse effects	37	5835.00	215,895.00
Blood savings	40	110.00	4400.00
Total		593,453.00	

**Table 3:** Clinical variables.

		Control n = 76	Study n = 135	Total n = 211	*p*-Value
Adverse events n (%)	Yes	40 (52.6%)	33 (25%)	73 (35.1%)	<0.001
	No	36 (47.4%)	102 (75%)	135 (64.9%)	
Blood requirements n (%)	Yes	19 (25%)	13 (9.9%)	32 (15.5%)	0.014
	No	57 (75%)	122 (90.1%)	179 (84.5%)	
Units of blood Mean (SD)		0.75 (2.24)	0.22 (0.80)	0.42 (1.52)	<0.001
Hospital Stay Mean (SD)		11.63 (10.63)	8.34 (6.70)	9.5 (8.4)	0.004
Re-admission n(%)	Yes	15 (19.7%)	10 (7.6%)	25 (12.1%)	0.014
	No	61 (80.3%)	125 (92.4%)	186 (87.9%)	

## Data Availability

The original contributions presented in this study are included in the article. Further inquiries can be directed to the corresponding author.

## Abbreviations

The following abbreviations are used in this manuscript:

APN	Advanced practice nurse
BIS	Body Image Scale
BMI	Body mass index
GERM	Grupo Español de Rehabilitación Multimodal
DRG	Diagnosis-related group
GLIM	Global Leadership Initiative on Malnutrition
IMPRICA	Implementación Nacional de la Vía RICA
